# Estimating epidemiological parameters of highly pathogenic avian influenza in common terns using exact Bayesian inference

**DOI:** 10.1111/1365-2656.70145

**Published:** 2025-10-08

**Authors:** David A. Ewing, Sandra Bouwhuis

**Affiliations:** ^1^ Biomathematics and Statistics Scotland Edinburgh UK; ^2^ Institute of Avian Research Wilhelmshaven Germany

**Keywords:** avian influenza, Bayesian inference, common tern, disease ecology, H5N1, MCMC

## Abstract

1. Highly pathogenic avian influenza (HPAI) is a contagious viral disease that has led to the culling of huge amounts of poultry as well as the mortality of countless wild birds. The recent panzootic that began in 2021 has been particularly notable for its devastating effect on seabird populations around the globe.
2. Whilst transmission of HPAI within poultry has been relatively well studied, the recency of the current panzootic, combined with data collection challenges, means that much less is known about key epidemiological parameters, such as reproduction numbers, $R_{0}$, of HPAI in wild populations.
3. We develop methodology to carry out exact Bayesian parameter inference using reversible jump Markov chain Monte Carlo applied to mortality data in the form of daily carcass counts over the duration of subsequent outbreaks in a colony of common terns, *Sterna hirundo*, in 2022 and 2023.
4. We estimate $R_{0}$ to be 3.7 (95% CI 2.3;7.2) in 2022, and 3.2 (95% CI 1.7;7.0) in 2023. The probability of mortality for an infected bird was estimated to drop from 0.26 (95% CI 0.24;0.28) in 2022 to 0.14 (95% CI 0.11;0.20) in 2023. Our findings furthermore suggest direct bird‐to‐bird transmission to be the predominant driver of infection within the colony, with environmental transmission playing a negligible role.
5. We interpret our results to suggest that daily carcass removal may have kept environmental transmission at bay and that increased immunity and/or a change of the strain of HPAI may have caused the drop in mortality, but that facilitating ‘social distancing’, for example by providing more suitable breeding habitat, such that breeding densities can be reduced, will be key to reduce disease transmission in colony‐breeding seabirds such as the terns.

## Graphical abstract

Statistical methodology was developed to understand key processes driving avian influenza in a seabird colony. Using common tern mortality data, it was shown that bird‐to‐bird transmission dominates environmental contamination, with mortality reducing between subsequent outbreaks in 2022 and 2023. Immunity and colony density were highlighted as factors shaping outbreak severity.

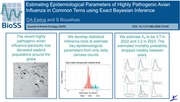

## 1 INTRODUCTION

Avian influenza is a viral disease that can cause substantial mortality in both domestic and wild birds. The current lineage of highly pathogenic avian influenza (HPAI, of type H5) emerged in 1996, with so‐called subclade 2.3.4.4 becoming dominant and spreading globally. This resulted in a panzootic responsible for the culling of half a billion poultry, as well as the infection of individuals of at least 60 species of mammals and 900 humans, and the mortality of wild birds across 356 recorded species (Klaassen & Wille, 2023; Kumar et al., 2024; Puryear & Runstadler, 2024; Wong, 2024). This panzootic is still expanding, with markedly more cases reported to the World Organisation for Animal Health since 2021 than at all dates prior to this time; even though accurate quantification of the level of mortality has not been possible due to severe underreporting of deaths, which is strongly and negatively linked to human population density (Klaassen & Wille, 2023). Nonetheless, the outbreak is known to have had disastrous effects on a wide range of species. For example, in the year 2022 alone, more than 20,000 sandwich tern carcasses were reported in Northwestern Europe (Knief et al., 2024), 1400 great skua and northern gannet corpses were documented on the Scottish island of Foula (Camphuysen & Gear, 2022), and an estimated 40,966 wild birds were reported sick or dead in Eastern Canada (Avery‐Gomm et al., 2024).

A notable feature of the recent HPAI panzootic is that it heavily affected seabirds (Cunningham et al., 2022; Falchieri et al., 2022; Pohlmann et al., 2023); a group of species half of which is listed as globally threatened with extinction or as near threatened (Phillips et al., 2023). This signals a change, as HPAI was previously observed to occur at very low prevalences in seabird populations, despite some species being exposed frequently (Lang et al., 2016). Most seabird species breed in dense colonies, where transmission is expected to be efficient, and their long lifespan makes their population dynamics sensitive to factors affecting adult survival (Sæther & Bakke, 2000). As such, understanding how avian influenza is transmitted within a seabird colony, and the rate at which transmission occurs, is crucial to understanding what, if anything, can be done to mitigate the spread (Boulinier, 2023). Doing so, however, is challenging because seabird colonies are often remote and hard to reach. Furthermore, biosecurity measures put in place during the outbreak, aimed at preventing accidental spread of the virus, hampered data collection efforts by restricting access to many colonies. In some cases, the use of drone imagery and machine learning approaches has helped to address these issues (Tyndall et al., 2024), but such cases are rare.

Mathematical and statistical modelling approaches are increasingly being used to support effective decision‐making for controlling and preventing avian influenza (Duan et al., 2023; Kirkeby & Ward, 2022). Advanced statistical inference methods provide us with the tools to estimate key epidemiological parameters, such as $R_{0}$ values, that tell us the number of secondary infections anticipated to stem from the introduction of a single infectious individual into a susceptible population at time t, even when data are sparse. These statistical tools are very flexible, enabling modelling assumptions to be tailored to match the underlying problem at hand and have been applied to tackle a range of disease challenges (Ewing et al., 2022; Neri et al., 2014; Nuismer et al., 2020; O'Hare et al., 2014). More specifically, a wide range of statistical approaches have been applied to estimate within‐ and between‐flock transmission parameters for HPAI in poultry farms (Kirkeby & Ward, 2022), often with a view to parameterising simulation models to aid decision makers in managing outbreaks (Institute (NL) et al., 2017). Similar efforts to estimate transmission in wild birds are more limited, likely due to limited data availability and funding. In a rare study of disease transmission within a seabird colony, Iverson et al. (2016) applied the likelihood‐based method of Forsberg‐White and Pagano (2008) to infer epidemic curves of mortality in a well‐studied common eider colony in the Canadian Arctic. They estimated changes in the reproductive number of avian cholera in a series of annual outbreaks, providing evidence for herd immunity to arise and mitigate mortality.

We adapt an exact Bayesian inference approach previously applied to estimate African swine fever transmission parameters within pig herds (Ewing et al., 2022) and apply this to estimate avian influenza transmission within a well‐studied colony of common terns at the Banter See in Germany over successive HPAI outbreaks in 2022 and 2023. Our model assumes disease transmission within the colony to follow a stochastic individual‐based epidemic model. We then infer key epidemiological parameters using data‐augmented reversible jump Markov chain Monte Carlo (RJ MCMC) requiring only daily recordings of carcasses. Finally, we highlight the wide applicability of the approach beyond the specific example presented here and interpret the results in the light of transmission modes and immunity, which are important for predicting long‐term effects on the population.

## 2 MATERIALS AND METHODS

### 2.1 Banter See common tern HPAI outbreak data

Data were collected during HPAI outbreaks in 2022 and 2023 at a breeding colony of common terns at the Banter See in Northern Germany. In 1992, 101 breeding adult terns of this colony were caught and marked with rings and transponders, and since 1992, all locally hatched birds have similarly been marked with a ring upon hatching as well as with a transponder shortly prior to fledging. The colony site consists of 6 concrete islands, each of which measures 10 by 5 m and is surrounded by a 60 cm wall. The walls support 44 platforms for terns to land on, and each platform is equipped with an antenna that reads transponder codes to record the (daily) presence of transponder‐marked individuals. During incubation, which is shared between male and female breeders, additional antennae are placed at each nest for 1–2 days to identify breeding individuals. Should ringed breeders not be identified by the antenna system (74 out of 788 breeders in 2022 and 41 out of 442 breeders in 2023), their rings are read. In the two outbreak years, this ring reading showed that 10 and 6 birds (i.e. 1% in both years) were immigrant breeders, whereas 64 and 35 breeders had a non‐functioning transponder (i.e. 8% in both years), respectively. Combined with 3‐times‐weekly checks of nests to record reproductive parameters and to mark offspring, these methods enable the systematic and remote documentation of individual presence and reproductive performance at the colony. Since 1992, the number of breeding pairs has ranged between 90 and 740, equalling 690, 340 and 215 in 2022, 2023 and 2024, respectively.

**FIGURE 1:**
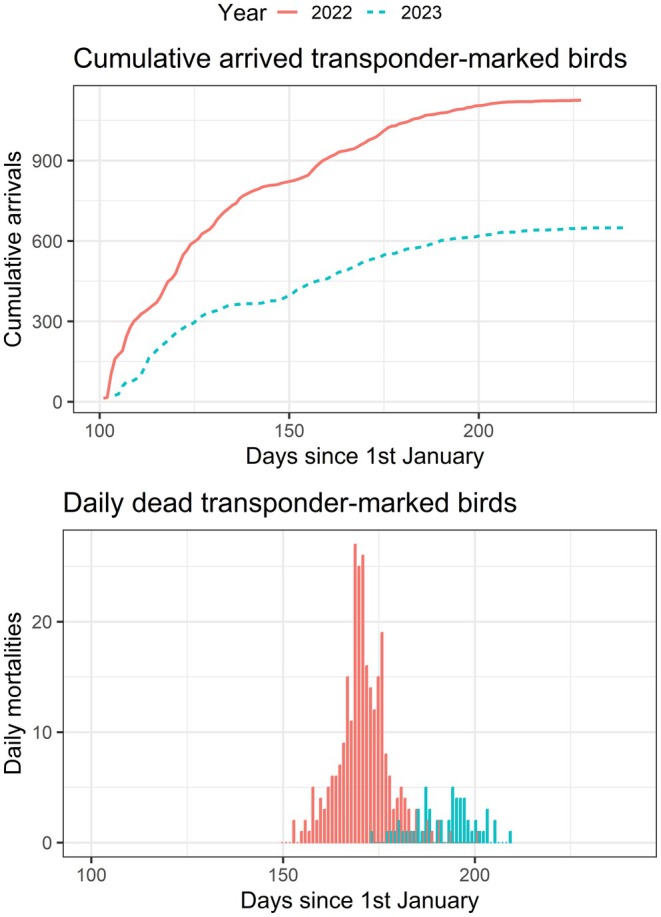
The cumulative number of transponder‐marked common terns registered daily at the Banter See colony (solid lines) is shown alongside the cumulative number of transponder‐marked birds found dead in the colony and its surrounding area (dashed lines) over the duration of the HPAI outbreaks in 2022 (left) and 2023 (right).

During the HPAI outbreak in 2022 and 2023, the colony and surrounding lake were monitored daily, and all birds found dead were removed from the environment and stored in a freezer at the field station. It is likely that some birds will have died sufficiently far from the colony that detection of dead birds will not be perfect, though it is expected that the proportion of the population that died and whose carcasses were not recovered will be low. Given that 26% of registered transponder‐marked birds were found dead in 2022 and the return rate in 2023 was 58%, this suggests that the maximum rate of non‐detection would be 16%, though it is highly likely to be lower than this because this would not account for mortality away from the colony in the intervening time due to, for example, HPAI at overwintering grounds, other mortality or birds skipping a breeding season. Similarly, in 2023, 9% of registered transponder‐marked birds were found dead, whilst the return rate in 2024 was 66%, leading to a maximum rate of non‐detection of 24%. This underdetection is accounted for in the inference approach. The total number of birds in the colony prior to mortality due to HPAI was estimated to be 2071 in 2022 and 1061 in 2023. The cumulative number of dead birds recorded within each year was 511 in 2022 and 111 in 2023, with the temporal trajectory of the subset of dead transponder‐marked bird detections (279 and 59 for 2022 and 2023, respectively) compared to the overall number of detected birds in each year shown in Figure 1. This work was performed under licences of the city of Wilhelmshaven (63‐04/03) and the Lower Saxony State Office for Consumer Protection and Food Safety (33.14‐42502‐04‐14/1392, 33.19‐42502‐04‐19/3068 and 33.12‐42502‐04‐24‐00555), Germany. The mortality data is available on Dryad (Ewing & Bouwhuis, 2025).

### 2.2 Model formulation and assumptions

We use the inference framework presented by Ewing et al. (2022), who developed exact Bayesian inference approaches to estimate epidemiological parameters of African swine fever virus (ASFV) from on‐farm mortality data early in outbreaks. The aim here is similar to the previous ASFV case because in both situations we seek to infer epidemiological parameters from only recordings of the number of dead animals observed each day over the monitoring period. There are, however, some important differences between the two scenarios that must be accounted for in the model framework and inference approach.

In contrast to a farm, a seabird colony is a largely unmanaged environment, meaning that the truncated observation window arising from the culling of animals following a disease diagnosis is not relevant here, and we can observe the entire duration of the outbreak. This opportunity to observe the whole outbreak makes accurate estimation of epidemiological parameters a more promising prospect; however, observation of a wild population comes with its own challenges. Firstly, we have no control over potential mixing between seabird species, and it is highly unlikely that we have perfect detection of all dead birds. Furthermore, unlike some strains of ASFV, which can reasonably be assumed to have a 100% mortality rate in domestic pigs, some proportion of seabirds will recover and develop immunity to avian influenza. This recovery means that, particularly in 2023, the initial population may comprise an unknown mix of susceptible and immune individuals.

We focus on modelling the spread of avian influenza within the colony and make the simplifying assumption that any mixing with other species has no impact on this transmission. Whilst this is clearly a limitation to the potential application of this methodology to other wild bird species or other colonies where there is substantial mixing, it is likely to be a reasonable assumption in the context of some populations, such as this one, which do not nest in close proximity with other colonies. There will be some interaction with birds from outside the colony when birds are foraging, but the opportunities for viral transmission when foraging at sea are likely to be negligible in comparison to transmission occurring within the colony when the birds are interacting in close proximity over the duration of a breeding season.

**FIGURE 2:**
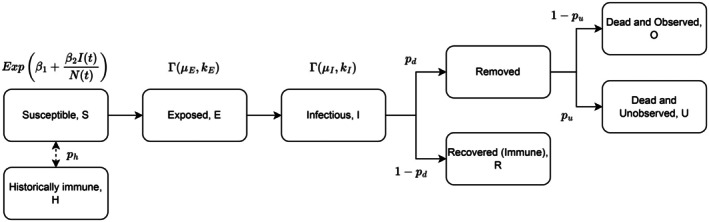
Model flowchart: Representation of the model, showing the progression of individuals from susceptible through the different infection states following exposure to HPAI. The historically immune individuals are not involved in disease transmission (beyond their contribution to the total population size, $N\left(t\right)$). A dashed (rather than solid) arrow links these immune individuals to the susceptible individuals to note that we infer the proportion of individuals in each group, but individuals do not transition from one state to another over the course of the outbreak.

The issues of imperfect detection of dead individuals and the inclusion of recovered immune individuals are addressed by extending the susceptible‐exposed‐infectious‐removed model framework to include the following set of categories: historically immune (H), susceptible (S), exposed (E), infectious (I), recovered (R), dead and observed (O) and dead and unobserved (U) (Figure 2). The initial population is split into historically immune and susceptible individuals with probability $p_{h}$ and $1-p_{h}$, respectively. Historically immune individuals are considered immune from having experienced an outbreak at the wintering grounds or in a previous year. Once susceptible individuals become infected, they enter the exposed state where they are infected but not infectious. After this latent period, individuals become infectious for an infectious period before either dying (with probability $p_{d}$) or recovering (with probability $1-p_{d}$). Those individuals that died are then observed (with probability $1-p_{u}$) or unobserved (with probability $p_{u}$). The historically immune individuals, $H\left(t\right)$, that acquired immunity from a previous outbreak and the recovered (immune) individuals that acquired immunity from the current outbreak, $R\left(t\right)$, are modelled separately. This allows us to infer parameters from outbreaks within a given year, rather than requiring that we explicitly model inter‐annual population dynamics. This is more feasible because not all birds are transponder‐marked (birds immigrating into the population generally are not caught and marked), making inclusion of the entire population in a multi‐year model very challenging.

We focus on estimation of $R_{0}$, though in the cases where it is assumed that some individuals in the population have prior immunity the more meaningful metric would perhaps be the effective reproduction number, $R_{e}$, which can be calculated as $R_{e}=R_{0}\left(1-p_{h}\right)$. This accounts for the reduction in onward transmission due to the reduced proportion of susceptible individuals. In cases where we assume the presence of prior immunity, we present both $R_{0}$ and $R_{e}$ values; however, we focus comparisons between years and between models on $R_{0}$ values both to ensure like‐for‐like comparisons and due to uncertainty around the levels of historical immunity (see Section 3.2).

Disease transmission within the colony is assumed to follow a stochastic individual‐based epidemic model. We assume a closed and homogeneously mixing population of size $N\left(t\right)$, $I\left(t\right)$ of whom are infectious at time t. At the start of the outbreak in each year we assume that $N\left(t\right)$ is the estimated total population size for the given year (2071 in 2022 and 1061 in 2023 as stated in Section 2.1), with $N\left(t\right)$ then decreasing over time in response to observed and inferred mortality due to HPAI. This assumes that the virus is not introduced by an early arrival and ignores the rate at which individuals will leave the colony after breeding or in response to infection (see the Discussion for a consideration of the potential impacts of these assumptions). Infection occurs either via direct frequency‐dependent within‐colony transmission, $\beta_{2}$ or via transmission from an environmental reservoir, $\beta_{1}$, such that the force of infection acting on susceptible individuals is given by $\lambda \left(t\right)=\left(\frac{\beta_{2}I\left(t\right)}{N\left(t\right)}+\beta_{1}\right)$. New infections therefore occur according to an inhomogeneous Poisson process with rate, $\lambda \left(t\right)$. By including the external force of infection we allow for the possibility that multiple individuals could have been infected by a process other than within‐colony transmission, thus implicitly allowing for the possibility that multiple introduction events into the colony could have occurred as in Ewing et al. (2022). This is consistent with pathogen sequence data that suggests multiple points of origin within this colony (2 in 2022, results not yet available for 2023). We also test the impact of our choice of frequency‐dependent transmission (as opposed to density‐dependent transmission) by considering a case where the $N\left(t\right)$ term is dropped from the denominator. For simplicity we do not attempt to explicitly model the environmental reservoir of infection but assume that it causes a constant background force of infection. We assume gamma distributions for the duration of the exposed and infectious periods.

The full set of model parameters is given by $\theta =\left(\beta_{1},\beta_{2},k_{L},\mu_{L},k_{I},\mu_{I},p_{d},p_{h},p_{u}\right)$. We simulate from this model using a modified Gillespie algorithm (Supporting Information S1).

### 2.3 Model likelihood and Bayesian inference

We use data‐augmented RJ MCMC to impute the unobserved exposure and infection times for observed dead birds alongside imputing full sets of exposure, infection and removal/recovery times for unobserved dead/recovered birds. Given that the complete history of event times for all individuals within the system can be expressed using the vectors e, i and r, where $e_{j}$, $i_{j}$ and $r_{j}$ represent the exposure, infection and recovery/death times, respectively, for individual j, we can write the likelihood as

(1) $$\begin{array}{ll} L\left(e,i,r|\theta \right)\propto & \left[\prod_{j\in E_{-w}}\left(\frac{\beta_{2}I\left(e_{j}^{-}\right)}{N\left(e_{j}^{-}\right)}+\beta_{1}\right)\right]\exp\left(-\int_{\min\left(e_{w}\right)}^{T}\left(\beta_{2}\frac{I\left(t\right)}{N\left(t\right)}+\beta_{1}\right)S\left(t\right)\mathrm{dt}\right)\\ & \prod_{j\in E}\;f_{\gamma }\left(r_{j}-i_{j};\mu_{I},k_{I}\right)\times f_{\gamma }\left(i_{j}-e_{j};\mu_{L},k_{L}\right)\\ & \prod_{j\in E}\;p_{d}^{n_{D}}{\left(1-p_{d}\right)}^{n_{R}}\prod_{j\in D}\;p_{u}^{n_{U}}{\left(1-p_{u}\right)}^{n_{O}}\\ & \prod_{j\in N}\;p_{h}^{n_{h}}{\left(1-p_{h}\right)}^{n_{0}-n_{h}}, \end{array}$$

where E denotes the set of all exposed individuals up to the time immediately following the final observed mortality $T_{f}$, w denotes the initial exposed individual in the population, $E_{-w}$ denotes the set of all exposed individuals excluding w, $e_{j}^{-}$ denotes the time immediately preceding the *j*th exposure, $e_{j}$ and $f_{\gamma }\left(x;\mu ,k\right)$ represents the gamma probability distribution with mean μ and shape parameter k. The second and third last product terms relate to the likelihood of the observations given the individuals' recovery and observation probabilities, whereby $n_{D}$, $n_{R}$, $n_{U}$ and $n_{O}$ give the number of dead, recovered, dead and undetected and dead and detected individuals respectively and D is the set of all dead individuals up to $T_{f}$. We also denote the number of susceptible and historically immune individuals by $n_{S}$ and $n_{H}$, respectively. The final product term gives the component of the likelihood relating to the probability that individuals begin the year as susceptible or historically immune, whereby $n_{h}$ is the number of individuals with historically acquired immunity and N is the set of all individuals in the population. Here, rather than $r^{+}$ referring to individuals which were infected but which would not have died until after the cull, we use $r^{+}$ to refer to individuals which either died but were unobserved or which recovered and so were not present in the observed mortality counts. We combine the prior and the likelihood into the posterior distribution

(2) $$p\left(\theta ,e,i,r^{+}|r^{-}\right)\propto L\left(e,i,r|\theta \right)p\left(\theta \right),$$

which summarises knowledge of the vector of unknown parameters, θ, the unknown recovery/removal times $e,i,r^{+}$, given the data $r^{-}$ (which stores the observed mortality times) and prior, $p\left(\theta \right)$, with dependence on the process model structure implicit.

The RJ MCMC algorithm follows the same steps as detailed by Ewing et al. (2022) (see Supporting Information S2 for full details). In short, we initialise by sampling parameter values from the priors and generating a set of exposure and infection times based on these parameters, we perform random walk Metropolis updates on each parameter, we then perform state‐space updates by changing exposure, infection and unobserved removal times, we separately perform a random walk Metropolis update on the initial infection time followed by a joint update on the initial infection time and all other non‐fixed event times and then we repeat these steps (excluding the initialisation) until the MCMC chain is well mixed and we have sufficient samples. Details of the possible parameter and state‐space updates accounting for recovery of infections, underdetection of mortalities and historically acquired immunity are detailed in Supporting Information S3. The chain tuning was carried out as per Ewing et al. (2022) (see Supporting Information S4). All simulations were coded in C++. MCMC chains were run on the UK's Crop Diversity Bioinformatics High Performance Computing (HPC) Linux cluster and took approximately 10 h to run for 3 million iterations for a colony of 1000 birds. Similar speeds should be possible on a sufficiently powerful desktop computer with sufficient cores to run the chains in parallel. Full code is available on GitHub (Ewing, 2025). In all cases, posterior samples were generated as follows; 4 chains of 3,000,000 iterations each were run with the first 10% of iterations discarded as burn‐in and every 100th iteration kept after that point. Potential scale reduction factors (PSRFs) were checked for convergence using the stableGR R function (Knudson & Vats, 2022) and values for all parameters were approximately 1 (Supporting Information S5) (Brooks & Gelman, 1998). Total effective sample sizes (ESSs) where calculated for each parameter using the coda library and were all found to be at least 150 (Table S1) (Gelman & Shirley, 2011; Plummer et al., 2024). Chains were also inspected visually for convergence. Deviance information criterion (DIC) values for parameter set, θ, were calculated as $0.5\mathrm{var}\left(D\right)+\text{mean}\left(D\right)$ where the deviance, D, is the chain of $-2\log\left(L\left(e,i,r|\theta \right)\right)$ (Spiegelhalter et al., 2002).

### 2.4 Priors

The true parameter values and the prior distributions for the simulation study are given in Table 1. Gamma distributions were used for the priors for all mean and shape parameters with estimated durations based on Kirkeby and Ward (2022). Uniform distributions were used for the transmission rates. Truncated beta distributions were used for the three probabilities. The truncation was necessary to avoid the chains converging to a state whereby all individuals that did not result in an observed mortality were assigned to the historically immune compartment. The same priors were used for the application to the Banter See data except for the priors for the probability an individual has historically acquired immunity, $p_{h}$, which were updated to $\mathrm{T\beta}\left(11,40,0.5\right)$ for 2022 and $\mathrm{T\beta}\left(21,150\right)$ for 2023. These priors were chosen to reflect the results of the antibody testing (detailed in Section 3.2) done prior to the outbreaks in each of these years.

**TABLE 1:** True parameter values and priors for the simulation study.

Parameter	Description	Value	Prior
$\mu_{L}$	Mean of the latent period distribution (days)	2	$\Gamma \left(2,10\right)$
$k_{L}$	Shape of the latent period distribution	5	$\Gamma \left(5,5\right)$
$\mu_{I}$	Mean of the infectious period distribution (days)	5	$\Gamma \left(5,10\right)$
$k_{I}$	Shape of the infectious period distribution	5	$\Gamma \left(5,5\right)$
$R_{0}$	Within‐colony transmission rate	5	$U\left(1,30\right)$
$\beta_{1}$	Background transmission rate	0.011000	$U\left(0,1\right)$
$p_{d}$	Probability an infectious individual dies	0.15	$\mathrm{T\beta}\left(2,6,0.5\right)$
		0.3	$\mathrm{T\beta}\left(2,6,0.5\right)$
$p_{u}$	Probability a dead individual is undetected	0.02	$\mathrm{T\beta}\left(4,200,0.1\right)$
$p_{h}$	Probability an individual has historically acquired immunity	0.1	$\mathrm{T\beta}\left(10,90,0.5\right)$
		0.3	$\mathrm{T\beta}\left(30,70,0.5\right)$

*Note*: Where multiple values were explored ($p_{d}$ and $p_{h}$), each row represents one of the values tested and the corresponding prior used. $\mathrm{T\beta}\left(\alpha ,\beta ,c\right)$ represents a truncated β distribution where the additional parameter, c, denotes the upper limit at which the distribution was truncated.

## 3 RESULTS

### 3.1 Simulation study

**FIGURE 3:**
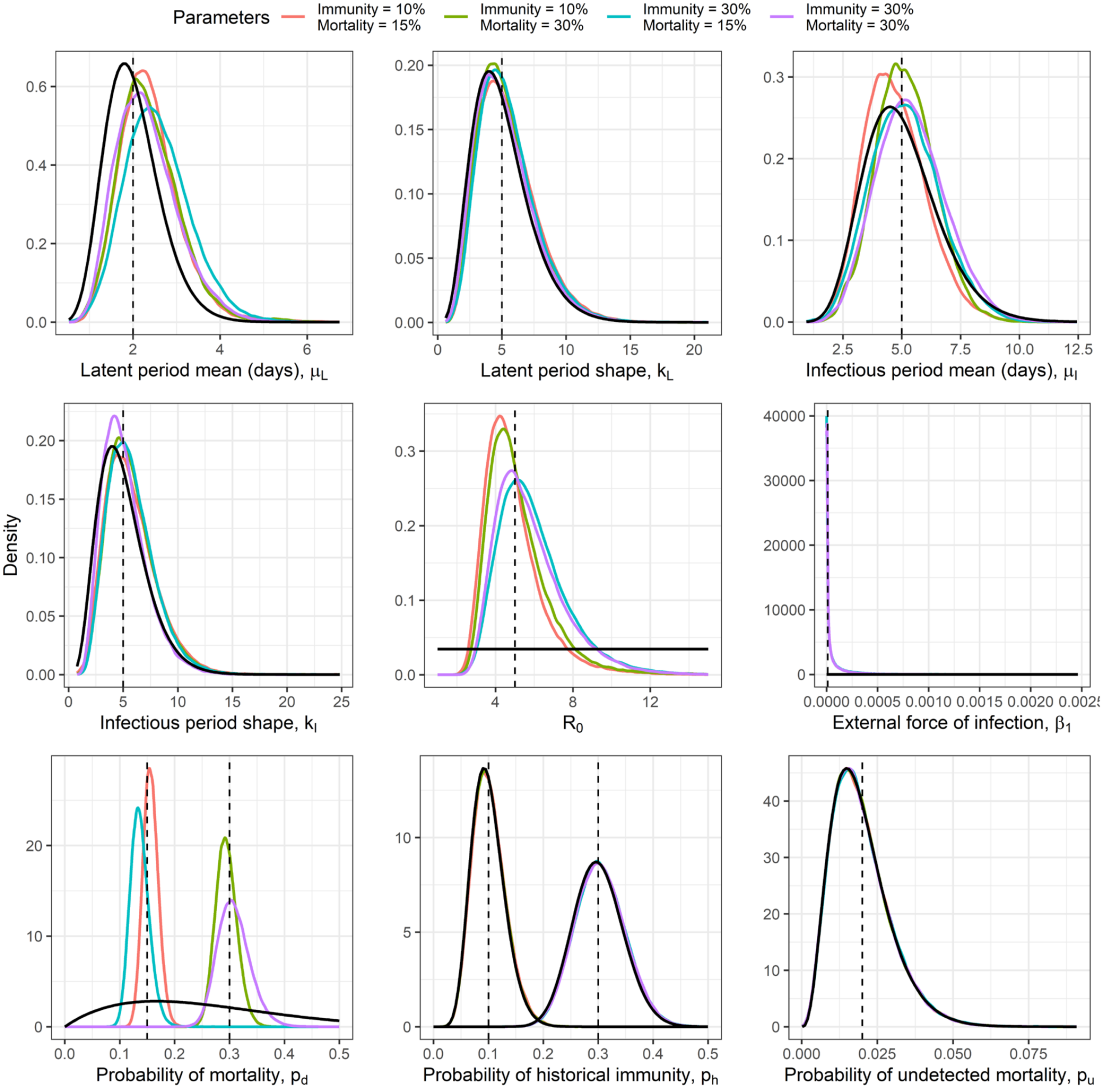
Posterior distributions of the parameters inferred based on the simulated data are shown by the coloured lines, with each colour representing a different simulated dataset. In the legend, immunity refers to the value for $p_{h}$, mortality to the value for $p_{d}$. The dashed vertical black lines show the true values. In the cases of $p_{h}$ and $p_{d}$, there are two true values representing the different true values considered across the four simulated datasets. The solid black lines show the prior distributions. In the case of $p_{h}$, two prior distributions are shown to represent that different priors were used for different true values. The priors and true values are also shown in Table 1. In cases where some lines appear to be missing, this is due to overplotting, as the distribution of the priors and posteriors match each other.

First, we simulated data to perform inference in a case where the true parameters were known, to assess the performance of the RJ MCMC algorithm in a situation mimicking that which generated the seabird data as closely as possible. We see from the results in Figure 3 that the posteriors closely map the priors for the latent and infectious period parameters, for the probability that birds have historically acquired immunity, $p_{h}$ and for the probability of failing to detect a dead bird, $p_{u}$. The requirement to provide informative priors for the latent and infectious period parameters is consistently the case for this model, and other similar models (Ewing et al., 2022), across the range of plausible parameter values, though these parameters can more readily be estimated and extrapolated from laboratory experiments than the others (Kirkeby & Ward, 2022). Inference is good for the transmission rate parameters and the probability that a bird dies of HPAI rather than recovers, $p_{d}$, with substantial improvement on the minimally informative priors for all the parameter combinations considered. When other less informative priors were tested for $p_{h}$, it was observed that the posterior did not deviate from the prior, such that informative priors are required to conduct robust and reliable inference. It is noteworthy that in the cases where historically acquired immunity took the lower of the two values, the estimated $R_{0}$ value was also estimated to be marginally lower, though in all cases the true value for $R_{0}$ was well within the 95% credible interval.

### 3.2 Application to common tern data

**FIGURE 4:**
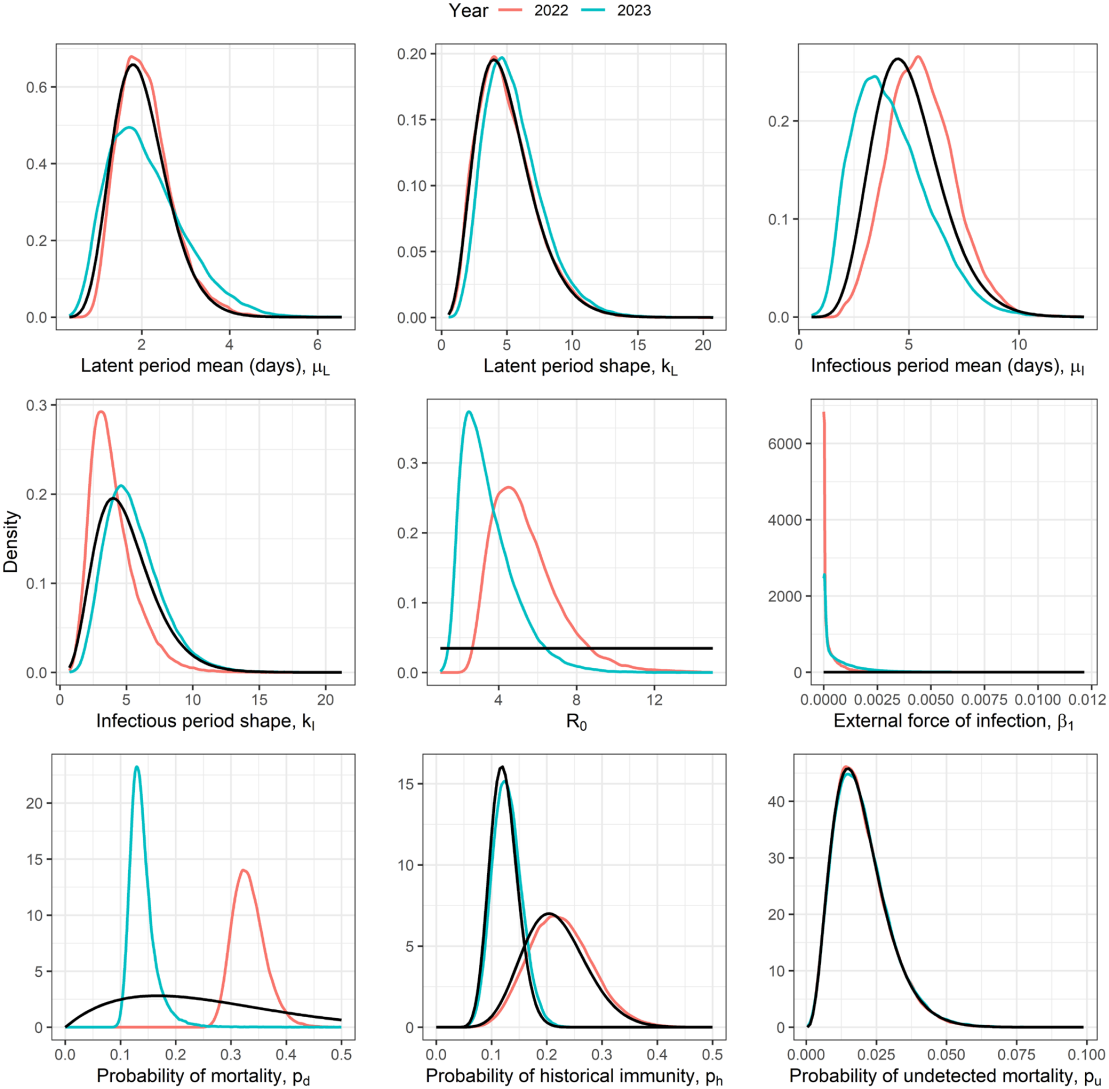
Posterior distributions of the parameters inferred based on the Banter See data are shown by the coloured lines. The solid black lines show the prior distributions. In the case of $p_{h}$, two prior distributions are shown, because the priors differed for the 2 years.

We applied the RJ MCMC algorithm to the data from the common tern colony at the Banter See for 2022 and 2023 separately. The posterior distributions are shown in Figure 4 and summaries given in Table 2. The estimated $R_{0}$ values were 5.1 (95% CI 3.0;9.7) in 2022 and 3.2 (95% CI 1.7;7.0) in 2023, suggesting a slight reduction from the first year to the second. The estimated $R_{e}$ values were 3.9 (95% CI 2.3;7.4) in 2022 and 2.8 (95% CI 1.5;6.1) in 2023. In both years, the external force of infection, $\beta_{1}$, was estimated to be very low, suggesting that almost all transmission within the colony was due to direct bird‐to‐bird transmission, rather than transmission from an environmental reservoir. The probability of an infectious bird dying from HPAI was estimated to be higher in 2022 (estimate = 0.33, 95% CI = 0.28;0.40) than in 2023 (estimate = 0.14, 95% CI = 0.11;0.20).

**TABLE 2:** The posterior median (and 95% credible intervals) are shown for each parameter for the full model and the model without historically acquired immunity in each of 2022 and 2023 separately along with the ΔDIC for each model relative to the model with the lowest DIC in that year.

Parameter	2022		2023	
	Full model	No historical immunity	Full model	No historical immunity
$\mu_{L}$	2.0	1.9	2.0	2.0
	(1.1; 3.5)	(1.0; 3.4)	(0.8; 4.1)	(0.8; 4.2)
$k_{L}$	4.7	4.9	5.2	5.2
	(1.7; 10.3)	(1.9; 10.5)	(2.2; 10.8)	(2.1; 10.8)
$\mu_{I}$	5.5	5.4	4.0	4.0
	(2.9; 8.8)	(3.0; 8.6)	(1.7; 8.0)	(1.8; 8.0)
$k_{I}$	3.6	4.0	5.2	5.2
	(1.6; 8.1)	(1.8; 8.8)	(2.3; 10.5)	(2.3; 10.4)
$R_{0}$	5.1	3.7	3.2	2.8
	(3.0; 9.7)	(2.3; 7.2)	(1.7; 7.0)	(1.5; 6.0)
$\beta_{1}N$	0.097	0.212	0.237	0.311
	(0.001; 2.921)	(0.001; 2.680)	(0.000; 3.509)	(0.000; 3.334)
$p_{d}$	0.33	0.26	0.14	0.12
	(0.28; 0.40)	(0.24; 0.28)	(0.11; 0.20)	(0.09; 0.17)
$p_{h}$	0.22	—	0.13	—
	(0.12; 0.35)		(0.08; 0.18)	
$p_{u}$	0.018	0.018	0.018	0.018
	(0.005; 0.042)	(0.005; 0.043)	(0.005; 0.043)	(0.005; 0.043)
Δ **DIC**	112,985	0	0	31,460

*Note*: We multiply $\beta_{1}$ by the colony size to facilitate comparison of the rates between years.

**FIGURE 5:**
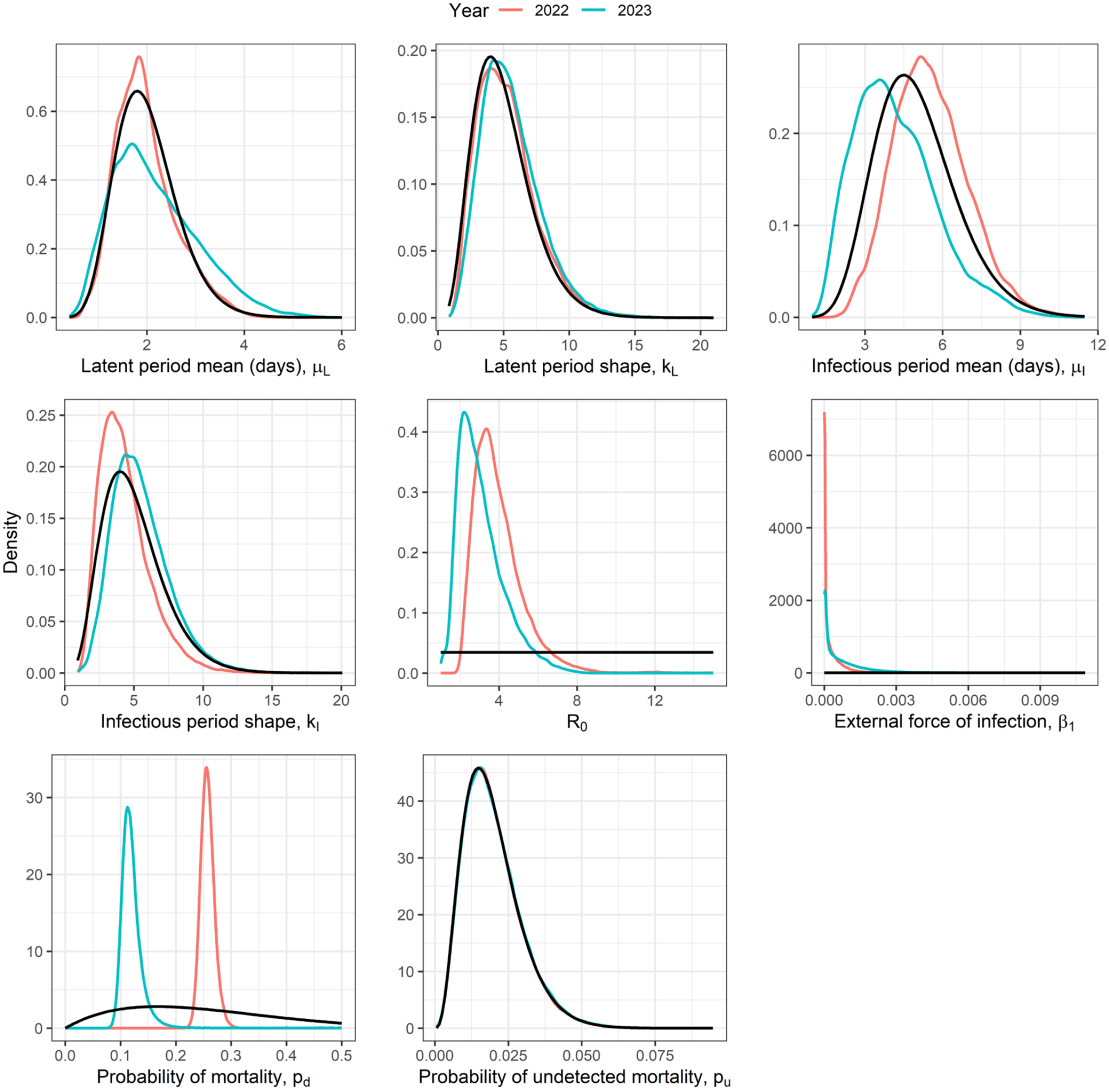
Posterior distributions of the parameters inferred based on the Banter See data under the assumption of no historically acquired immunity are shown by the coloured lines. The solid black lines show the prior distributions.

These results were obtained under a, perhaps counterintuitive, prior belief that the degree of historically acquired immunity was higher in 2022 than in 2023. This was based on serological sampling conducted prior to each outbreak, that found general antibodies against avian influenza in 14 of 62 (23%) sampled individuals prior to the outbreak in 2022, and 27 of 223 (12%) sampled individuals prior to the outbreak in 2023. It is, however, unknown to what extent these antibodies may confer immunity to H5N1 specifically (and there wasn't enough serum to test this in the samples of 2022), such that we compare these findings against a case where the probability of historical immunity was set to zero in each year. Figure 5 shows that the only parameters that are notably affected by the inclusion of historically acquired immunity are $R_{0}$ and the probability of mortality in an infected individual, $p_{d}$. The effect on $R_{0}$ is relatively modest, with slight reductions from 5.1 to 3.7 (95% CI 2.3;7.2) in 2022, and from 3.2 to 2.8 (95% CI 1.5;6.0) in 2023. The probability of mortality of infected individuals is not notably different in 2023, with an estimated decrease in the probability of just 0.02. The impact is more notable in 2022, however, due to the higher proportion of individuals with antibodies prior to this outbreak, resulting in an estimate for $p_{d}$ of 0.26 (down from 0.33), with 95% CI from 0.24 to 0.28.

The impact of the priors chosen for the latent and infectious period parameters was explored and it was found that the estimates of $R_{0}$ and $p_{d}$ were likely to be robust to minor mis‐specification of these priors; however, it should be noted that assuming a shorter latent period slightly decreased the estimated $R_{0}$ values, whilst assuming a longer infectious period is likely to have the opposite effect. For a full discussion see Supporting Information S6. The impact of the assumed prior distribution on the probability that a carcass was undetected, $p_{u}$, was also explored and found to have negligible impact on the results within the range of plausible values (Supporting Information S7).

**FIGURE 6:**
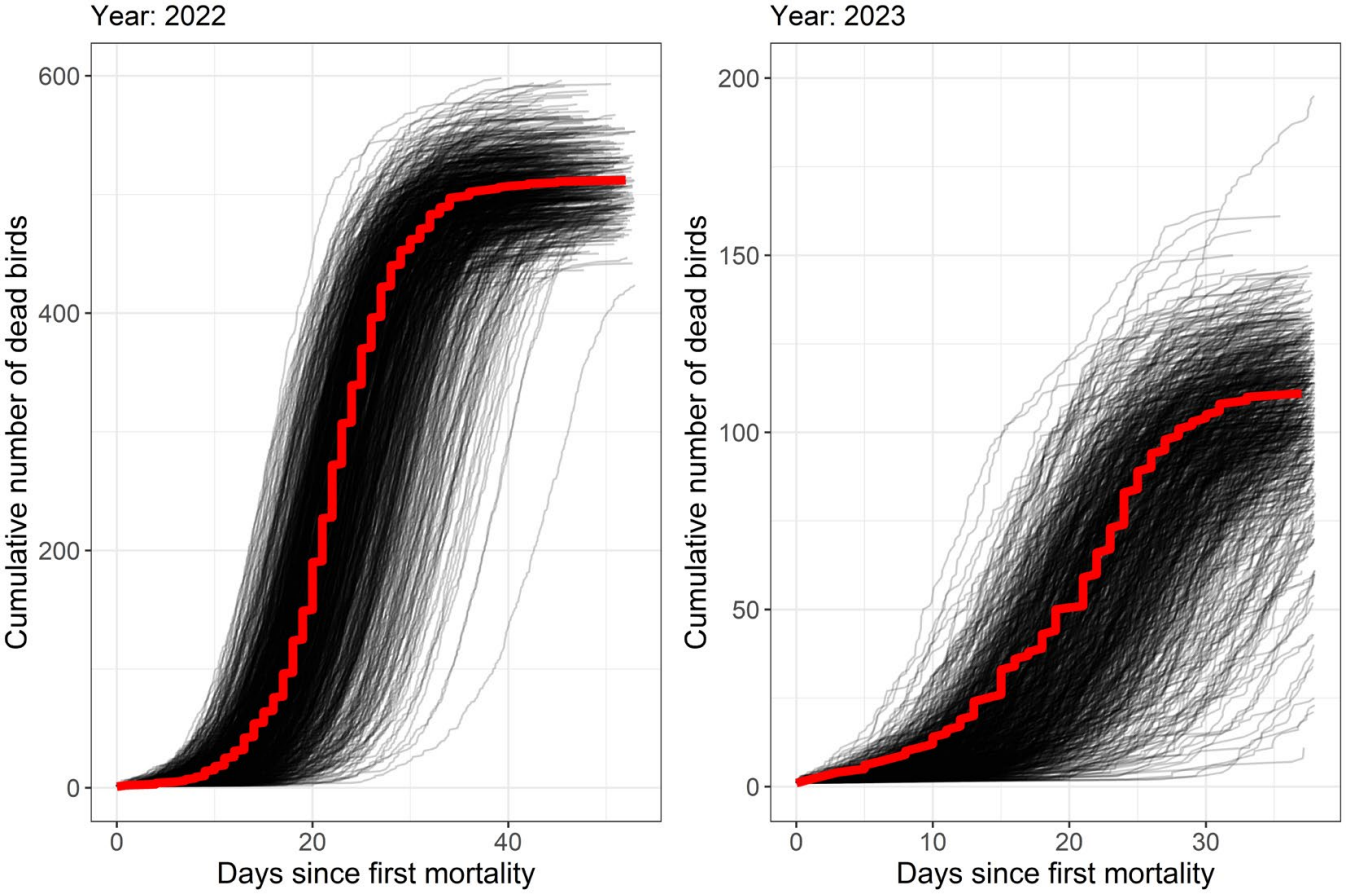
Posterior predictive checks for 2022 and 2023. The grey lines show simulated trajectories of the outbreaks given values sampled from the MCMC chains. The red lines show the observed mortality curve for each year.

The posterior predictive checks (shown for the case assuming historically acquired immunity in Figure 6) show good agreement between the observed data and the datasets simulated based on values drawn from the converged MCMC chains, suggesting that the fitted parameter values are plausible for having generated the observed outbreaks. Results of these checks looked very similar in the case without historically acquired immunity. We compared the deviance information criteria (DIC) values of the models both with and without historically acquired immunity for each of the years to understand whether a particular model appeared more appropriate than the other. In 2022, during the first outbreak, we observed that the model without historically acquired immunity performed better (Table 2). In 2023, however, we observed the opposite, with the inclusion of historically acquired immunity resulting in an improvement of the DIC score. This is consistent with what we would have expected given that the first outbreak occurred in 2022, though it appears inconsistent with the results of the antibody testing. As such, it seems likely that the antibodies detected in 2022 were not predictive of immunity to H5N1, whereas those in 2023 were. Given these results, we consider the most reliable estimates to be those without historically acquired immunity in 2022 and those with historically acquired immunity in 2023 and thus present these in the abstract and discussion sections.

## 4 DISCUSSION

We have developed an approach to generate robust estimates of key epidemiological parameters that describe HPAI transmission within a seabird colony during a 2‐year outbreak. We estimate that the $R_{0}$ values in 2022 (3.7, 95% CI 2.3;7.2) and 2023 (3.2, 95% CI 1.7;7.0) were similar, with only a very slight reduction between years. The probability of mortality in infected birds decreased more notably, from 0.26 (95% CI 0.24;0.28) in 2022 to 0.14 (95% CI 0.11;0.20) in 2023, with no overlap between the 95% credible intervals for the 2 years. We also estimate that the majority of infections was due to direct bird‐to‐bird transmission rather than exposure via an environmental reservoir, potentially suggesting that the prompt removal of carcasses may have alleviated the risk of environmental transmission. These are some of the first findings that quantify key transmission parameters of HPAI within a seabird colony and provide valuable information to begin to understand potential long‐term effects of HPAI outbreaks on already vulnerable seabird populations and to guide and inform future conservation efforts.

Generation of our estimates only required the number of carcasses reported each day over the duration of the outbreak and informative priors on the latent and infectious periods of the virus, highlighting the wide applicability of this methodology across different wildlife disease systems where data collection is challenging (Stallknecht, 2007). Our estimates were reasonably robust to assumptions about the duration of the latent and infectious periods of the virus, the assumed level of prior immunity in the population and the assumed level of underdetection of birds that had died.

The estimates of $R_{0}$ presented here are slightly higher than those reported by Kirkeby and Ward (2022), who found a median estimate for H5N1 of 2.18 with an interquartile range of 0.99−3.40 across 40 studies on commercial poultry farms. This makes sense, given that a commercial poultry unit is a much more strictly managed environment, aimed at reducing contacts between animals and thereby reducing transmission. We are not aware of studies in other seabird colonies to allow for a direct comparison.

We estimate the reproduction rate of the virus to have decreased from 2022 to 2023 by approximately 10%–15%, whilst the proportion of infected birds that died decreased by approximately 55%–60%. One reason for these decreases could be the development of immunity, although another could be a difference in the strain of the virus that was introduced in the 2 years, with the 2023 strain perhaps being less virulent and less severe. In addition, it is known that demographic changes occurred in the colony between the 2 years, because the outbreak in 2022 resulted in mortality skewed towards older birds and breeders, compared to younger birds and non‐breeders. This could result in the 2023 outbreak appearing to be less transmissible and to lead to lower mortality due to the higher resilience of the population. Finally, the colony was estimated to be approximately half the size in 2023 (340 breeding pairs) compared to 2022 (690 breeding pairs) such that the density of nests was lower, which may have reduced transmission. It is unclear whether density‐ or frequency‐dependent transmission is most likely for this colony, and in reality, it is likely that the most appropriate form may lie somewhere in between. Numerous studies have shown that the choice of transmission mode can have profound impacts when modelling disease and parasite transmission (Borremans et al., 2017; Hopkins et al., 2020; Smith et al., 2009). Consequently, one could hypothesise that a more thorough accounting of the changing density and contact rates may shed light on the difference in transmission rates and may point towards dose‐dependent effects on the severity of symptoms and disease outcomes.

We assume that the initial population at the start of each season, $n_{0}$, was the total estimated population size for that year, and thus that return of all birds to the colony was synchronous, i.e. the virus was not introduced by an early arrival to the colony when the total population size was below $n_{0}$. We also did not model the rate at which birds may have left the colony as, although there is evidence that this rate can be influenced by an HPAI outbreak (Jeglinski et al., 2024), the rate of departure is less well known than the rate of arrival. The result of these assumptions is that the population size in our inference algorithm started at $n_{0}$ (the total population size) when the outbreak began, and decreased through time only due to HPAI‐induced mortality. In 2022, approximately 70% of recorded birds had returned by the time at which the first mortality was recorded (Figure 1, upper panel, red line), whilst in 2023 approximately 80% of birds had returned by this point (Figure 1, upper panel, blue line). As such, we consider the assumption that the initial population size was the total population size to unlikely be influential. Furthermore, the period of exponential increase in HPAI cases, where the information to estimate $R_{0}$ values lies, lasted 3–4 weeks (Figure 1) and the change in population size over this time period driven by late arrivals and early departures is likely to be relatively small. This combination of factors meant that we found the choice of transmission mode was not influential in our analyses (Supporting Information S8), although future model developments could look to relax these assumptions.

A more promising area for further development of the inference approach may be in the explicit inclusion of spatial structure in disease transmission, which was considered beyond the scope of the analyses presented here. Typically, analyses of the spatiotemporal spread of HPAI have focused on spread between, rather than within, colonies or flocks of wild or domestic populations (Kirkeby & Ward, 2022). The common terns at the Banter See breed on a series of six artificial, rectangular platforms and the spatial location of the nests is known for the subset of transponder‐marked breeders. Previous analyses have shown that nests exhibit a regular spatial pattern, suggesting that birds seek to maximize the distance between their chosen nest site and that of conspecifics that arrived earlier (Bouwhuis et al., 2020). Towards the end of the breeding season, the time of departure is then related to the time of arrival. It may be that the rate of transmission and extent of infection would be strongly influenced by the location of introduction (a central platform versus an end platform) and the timing of introduction (an early versus a late arrival). Given that we observe that infection appears to be driven by bird‐to‐bird transmission rather than environmental transmission, a spatial model of transmission could refine the estimates we present.

We have shown that the exact Bayesian inference approach employed here can be flexible, having adapted it from the original application to African swine fever transmission on a pig farm (Ewing et al., 2022) to understand HPAI transmission in a seabird colony. This flexibility and applicability to situations where data is sparse (e.g. only mortality counts are recorded) is a particular strength when seeking to infer epidemiological parameters from wild populations, where data collection opportunities are constrained and the epidemiological systems will vary. Identification of further datasets to which this, or similar, methodologies could be applied to improve understanding of avian influenza transmission across a range of colonies and a range of species should be a priority to better understand the impact of the current panzootic on wild bird populations. Methods could also be further developed to investigate epidemiological parameters where mortality counts are available at lower temporal resolution, but in conjunction with auxiliary information, such as carcass scoring, to estimate the time of mortality (Camphuysen & Gear, 2022). These models and fitted parameter values could also be integrated into larger scale spatiotemporal models to estimate wider HPAI risk and impact (Boulinier, 2023; McDuie et al., 2024).

Overall, for the terns, we conclude our analyses to suggest that the daily removal of carcasses may have kept environmental transmission of HPAI to a minimum, whilst the build‐up of immunity may have only been slow, such that ‘social distancing’ may be key to reducing disease transmission and preventing further population declines. This could be achieved when breeding densities would be reduced in response to the offering of more suitable breeding habitat, making up for recent habitat loss.

## AUTHOR CONTRIBUTIONS

David A. Ewing: Conceptualization; formal analysis; investigation; methodology; software; validation; visualisation; writing—original draft; writing—review and editing. Sandra Bouwhuis: Conceptualization; data curation; investigation; methodology; validation; writing—review and editing.

## CONFLICT OF INTEREST STATEMENT

The authors have no conflicts of interest to disclose.

## STATEMENT ON INCLUSION

Our study brings together authors from two different countries, including one scientist based in the country where the study was carried out. All authors were engaged early on with the research and study design to ensure that the diverse sets of perspectives they represent were considered from the onset. Whenever relevant, literature published by scientists from the region was cited; efforts were made to consider relevant work published in the local language.

## Supporting information


**Table S1.** The total PSRF values and total ESSs are shown for each parameter for the model without historically acquired immunity in 2022 and the model with historically acquired immunity in 2023. ’‐’ denotes that no value was calculated for this entry.
**Table S2.** The posterior median (and 95% credible intervals) are shown for each parameter for the model with the “baseline” priors (main text Table 1), a prior giving a longer mean infectious period (*μ_I_* ~ Γ(7, 10)), a prior giving smaller values to the shape parameters on the latent and infectious periods (*k_L_*, *k_I_* ~ Γ(2.5, 5)), and a prior giving a shorter latent period (*μ_L_* ~ Γ(1, 10)). Results are shown for 2022 (top) and 2023 (bottom) separately with the DIC for each model. We multiply β1 by the colony size to facilitate comparison of the rates between years.
**Figure S1.** The posterior distributions of the parameters inferred based on the Banter See data are shown by the coloured lines. The solid black lines showthe prior distributions. In the case of ph two prior distributions are shown because the priors differed for the two years. The solid coloured lines show estimates assuming the baseline level of underdetection given by the prior in Table 1 and the dashed lines show estimates based on a prior belief of a higher level of underdetection given by Tβ(11, 200, 0.16).
**Figure S2.** The posterior distributions of the parameters inferred based on the Banter See data are shown by the coloured lines. The solid black lines showthe prior distributions. In the case of ph two prior distributions are shown because the priors differed for the two years. The solid coloured lines show estimates assuming density dependent transmission and the dashed lines show estimates assuming frequency dependent transmission.

## Data Availability

Data available from the Dryad Digital Repository https://doi.org/10.5061/dryad.ncjsxkt8g (Ewing & Bouwhuis, 2025). Code available from GitHub.

## References

[jane70145-bib-0001] Avery‐Gomm, S. , Barychka, T. , English, M. , Ronconi, R. A. , Wilhelm, S. I. , Rail, J.‐F. , Cormier, T. , Beaumont, M. , Bowser, C. , Burt, T. V. , Collins, S. M. , Duffy, S. , Giacinti, J. A. , Gilliland, S. , Giroux, J.‐F. , Gjerdrum, C. , Guillemette, M. , Hargan, K. E. , Jones, M. , … Wight, J. (2024). Wild bird mass mortalities in eastern Canada associated with the highly pathogenic avian influenza A (H5N1) virus, 2022. Ecosphere, 15, e4980.

[jane70145-bib-0002] Borremans, B. , Reijniers, J. , Hens, N. , & Leirs, H. (2017). The shape of the contact–density function matters when modelling parasite transmission in fluctuating populations. Royal Society Open Science, 4(11), 171308. 10.1098/rsos.171308 29291115 PMC5717690

[jane70145-bib-0003] Boulinier, T. (2023). Avian influenza spread and seabird movements between colonies. Trends in Ecology & Evolution, 38(5), 391–395. 10.1016/j.tree.2023.02.002 36841680

[jane70145-bib-0004] Bouwhuis, S. , Ballani, F. , Bourgeois, M. , & Stoyan, D. (2020). Colony size affects breeding density, but not spatial distribution type, in a seabird. Behavioral Ecology, 31(5), 1113–1119. 10.1093/beheco/araa058

[jane70145-bib-0005] Brooks, S. P. , & Gelman, A. (1998). General methods for monitoring convergence of iterative simulations. Journal of Computational and Graphical Statistics, 7(4), 434–455.

[jane70145-bib-0006] Camphuysen, C. , & Gear, S. (2022). Great Skuas and northern gannets on Foula, summer 2022—An unprecedented, H5N1 related massacre field Report . 10.25850/nioz/7b.b.gd

[jane70145-bib-0007] Cunningham, E. J. A. , Cunningham, E. , Gamble, A. , Hart, T. , Humphreys, E. , Philip, E. , Tyler, G. , & Wood, M. (2022). The incursion of highly pathogenic avian influenza (HPAI) into North Atlantic seabird populations: An interim report from the 15th international seabird group conference. Seabird, 34, 67–73. 10.61350/sbj.34.67

[jane70145-bib-0008] Duan, C. , Li, C. , Ren, R. , Bai, W. , & Zhou, L. (2023). An overview of avian influenza surveillance strategies and modes. Science in One Health, 2, 100043. 10.1016/j.soh.2023.100043 39077039 PMC11262264

[jane70145-bib-0009] Ewing, D. A. (2025). HPAI *inference for common terns*. Version v1.0.0. 10.5281/zenodo.17054917

[jane70145-bib-0010] Ewing, D. A. , & Bouwhuis, S. (2025). Estimating epidemiological parameters of highly pathogenic avian influenza in common terns using exact Bayesian inference. *Dryad Digital Repository*, 10.5061/dryad.ncjsxkt8g PMC12673236 41063438

[jane70145-bib-0011] Ewing, D. A. , Pooley, C. M. , Gamado, K. M. , Porphyre, T. , & Marion, G. (2022). Exact Bayesian inference of epidemiological parameters from mortality data: Application to African swine fever virus. Journal of the Royal Society Interface, 19(188), 20220013. 10.1098/rsif.2022.0013 35259955 PMC8905154

[jane70145-bib-0012] Falchieri, M. , Reid, S. M. , Ross, C. S. , James, J. , Byrne, A. M. P. , Zamfir, M. , Brown, I. H. , Banyard, A. C. , Tyler, G. , Philip, E. , & Miles, W. (2022). Shift in HPAI infection dynamics causes significant losses in seabird populations across Great Britain. Veterinary Record, 191(7), 294–296. 10.1002/vetr.2311 36205958

[jane70145-bib-0013] Forsberg‐White, L. , & Pagano, M. (2008). A likelihood‐based method for real‐time estimation of the serial interval and reproductive number of an epidemic. Statistics in Medicine, 27, 2999–3016. 18058829 10.1002/sim.3136 PMC3951165

[jane70145-bib-0014] Gelman, A. , & Shirley, K. (2011). Inference from simulations and monitoring convergence. In Handbook of Markov chain Monte Carlo (pp. 162–174). Chapman and Hall/CRC.

[jane70145-bib-0015] Hopkins, S. R. , Fleming‐Davies, A. E. , Belden, L. K. , & Wojdak, J. M. (2020). Systematic review of modelling assumptions and empirical evidence: Does parasite transmission increase nonlinearly with host density? Methods in Ecology and Evolution, 11(4), 476–486. 10.1111/2041-210X.13361

[jane70145-bib-0016] Institute (NL) , Central Veterinary , de Koeijer, A. , Arnold, M. , Gonzales, J. , & Jan Boender, G. (2017). Data analysis and predictive modelling of HPAI H5 and H7 outbreaks in the EU 2005‐2015. EFSA Supporting Publications, 14(10), 1285E. 10.2903/sp.efsa.2017.EN-1285

[jane70145-bib-0017] Iverson, S. A. , Gilchrist, H. G. , Soos, C. , Buttler, I. I. , Harms, N. J. , & Forbes, M. R. (2016). Injecting epidemiology into population viability analysis: Avian cholera transmission dynamics at an Arctic seabird Colony. Journal of Animal Ecology, 85(6), 1481–1490. 10.1111/1365-2656.12585 27548394

[jane70145-bib-0018] Jeglinski, J. , Jeglinski, J. W. E. , Lane, J. V. , Votier, S. C. , Furness, R. W. , Hamer, K. C. , McCafferty, D. J. , Nager, R. G. , Sheddan, M. , Wanless, S. , & Matthiopoulos, J. (2024). HPAIV outbreak triggers short‐term colony connectivity in a seabird metapopulation. Scientific Reports, 14, 3126. 10.1038/s41598-024-53550-x 38326368 PMC10850054

[jane70145-bib-0019] Kirkeby, C. , & Ward, M. P. (2022). A review of estimated transmission parameters for the spread of avian influenza viruses. Transboundary and Emerging Diseases, 69(6), 3238–3246. 10.1111/tbed.14675 35959696 PMC10088015

[jane70145-bib-0020] Klaassen, M. , & Wille, M. (2023). The plight and role of wild birds in the current bird flu Panzootic. Nature Ecology & Evolution, 7(10), 1541–1542. 10.1038/s41559-023-02182-x 37587226

[jane70145-bib-0021] Knief, U. , Bregnballe, T. , Alfarwi, I. , Ballmann, M. Z. , Brenninkmeijer, A. , Bzoma, S. , Chabrolle, A. , Dimmlich, J. , Engel, E. , Fijn, R. , Fischer, K. , Hälterlein, B. , Haupt, M. , Hennig, V. , Herrmann, C. , in ‘t Veld, R. , Kirchhoff, E. , Kristersson, M. , Kühn, S. , … Courtens, W. (2024). Highly pathogenic avian influenza causes mass mortality in Sandwich tern *Thalasseus Sandvicensis* breeding colonies across North‐Western Europe. Bird Conservation International, 34, e6. 10.1017/S0959270923000400

[jane70145-bib-0022] Knudson, C. , & Vats, D. (2022). stableGR: A stable Gelman‐Rubin diagnostic for Markov chain Monte Carlo .

[jane70145-bib-0023] Kumar, P. , Sharma, A. , Apostolopoulos, V. , Gaidhane, A. M. , & Satapathy, P. (2024). Australia's first human case of H5N1 and the current H7 poultry outbreaks: Implications for public health and biosecurity measures. The Lancet Regional Health—Western Pacific, 48, 101141. 10.1016/j.lanwpc.2024.101141 39119239 PMC11305885

[jane70145-bib-0024] Lang, A. S. , Lebarbenchon, C. , Ramey, A. M. , Robertson, G. J. , Waldenström, J. , & Wille, M. (2016). Assessing the role of seabirds in the ecology of influenza A viruses. Avian Diseases, 60(1 Suppl), 378–386. 10.1637/11135-050815-RegR 27309082

[jane70145-bib-0025] McDuie, F. , Overton, C. T. , Lorenz, A. A. , Matchett, E. L. , Mott, A. L. , Mackell, D. A. , Ackerman, J. T. , De La Cruz, S. E. W. , Patil, V. P. , Prosser, D. J. , Takekawa, J. Y. , Orthmeyer, D. L. , Pitesky, M. E. , Díaz‐Muñoz, S. L. , Riggs, B. M. , Gendreau, J. , Reed, E. T. , Petrie, M. J. , Williams, C. K. , … Casazza, M. L. (2024). Mitigating risk: Predicting H5N1 avian influenza spread with an empirical model of bird movement. Transboundary and Emerging Diseases, 2024(1), 5525298. 10.1155/2024/5525298 40303041 PMC12016750

[jane70145-bib-0026] Neri, F. M. , Cook, A. R. , Gibson, G. J. , Gottwald, T. R. , & Gilligan, C. A. (2014). Bayesian analysis for inference of an emerging epidemic: Citrus canker in urban landscapes. PLoS Computational Biology, 10(4), 1–16. 10.1371/journal.pcbi.1003587 PMC3998883 24762851

[jane70145-bib-0027] Nuismer, S. L. , Remien, C. H. , Basinski, A. J. , Varrelman, T. , Layman, N. , Rosenke, K. , Bird, B. , Jarvis, M. , Barry, P. , Hanley, P. W. , & Fichet‐Calvet, E. (2020). Bayesian estimation of Lassa virus epidemiological parameters: Implications for spillover prevention using wildlife vaccination. PLoS Neglected Tropical Diseases, 14(9), e0007920. 10.1371/journal.pntd.0007920 32956349 PMC7529244

[jane70145-bib-0028] O'Hare, A. , Orton, R. J. , Bessell, P. R. , & Kao, R. R. (2014). Estimating epidemiological parameters for bovine tuberculosis in British cattle using a Bayesian partial‐likelihood approach. Proceedings of the Royal Society B: Biological Sciences, 281(1783), 20140248. 10.1098/rspb.2014.0248 PMC3996616 24718762

[jane70145-bib-0029] Phillips, R. , Fort, J. , & Dias, M. P. (2023). Conservation status and overview of threats to seabirds. In Conservation of marine birds (pp. 33–56). Academic Press. 10.1016/B978-0-323-88539-3.00015-7

[jane70145-bib-0030] Plummer, M. , Best, N. , Cowles, K. , Vines, K. , Sarkar, D. , Bates, D. , Almond, R. , & Magnusson, A. (2024). Coda: Output analysis and diagnostics for MCMC. CRAN.

[jane70145-bib-0031] Pohlmann, A. , Stejskal, O. , King, J. , Bouwhuis, S. , Packmor, F. , Ballstaedt, E. , Hälterlein, B. , Hennig, V. , Stacker, L. , Graaf, A. , Hennig, C. , Günther, A. , Liang, Y. , Hjulsager, C. , Beer, M. , & Harder, T. (2023). Mass mortality among colony‐breeding seabirds in the German Wadden Sea in 2022 due to distinct genotypes of HPAIV H5N1 clade 2.3.4.4b. Journal of General Virology, 104(4), 001834. 10.1099/jgv.0.001834 37014781

[jane70145-bib-0032] Puryear, W. B. , & Runstadler, J. A. (2024). High‐pathogenicity avian influenza in wildlife: A changing disease dynamic that is expanding in wild birds and having an increasing impact on a growing number of mammals. Journal of the American Veterinary Medical Association, 262, 601–609. 10.2460/javma.24.01.0053 38599231

[jane70145-bib-0033] Sæther, B.‐E. , & Bakke, Ø. (2000). Avian life history variation and contributions of demographic traits to the population growth rate. Ecology, 81(3), 642–653. 10.1890/0012-9658(2000)081[0642:ALHVAC]2.0.CO;2

[jane70145-bib-0034] Smith, M. J. , Telfer, S. , Kallio, E. R. , Burthe, S. , Cook, A. R. , Lambin, X. , & Begon, M. (2009). Host–pathogen time series data in wildlife support a transmission function between density and frequency dependence. Proceedings of the National Academy of Sciences of the United States of America, 106(19), 7905–7909. 10.1073/pnas.0809145106 19416827 PMC2672915

[jane70145-bib-0035] Spiegelhalter, D. J. , Best, N. G. , Carlin, B. P. , & Van Der Linde, A. (2002). Bayesian measures of model complexity and fit. Journal of the Royal Statistical Society, Series B: Statistical Methodology, 64(4), 583–639. 10.1111/1467-9868.00353

[jane70145-bib-0036] Stallknecht, D. E. (2007). Impediments to wildlife disease surveillance, research, and diagnostics. In J. E. Childs , J. S. Mackenzie , & J. A. Richt (Eds.), Wildlife and emerging zoonotic diseases: The biology, circumstances and consequences of cross‐species transmission (pp. 445–461). Springer Berlin Heidelberg. 10.1007/978-3-540-70962-6_17 17848074

[jane70145-bib-0037] Tyndall, A. A. , Nichol, C. J. , Wade, T. , Pirrie, S. , Harris, M. P. , Wanless, S. , & Burton, E. (2024). Quantifying the impact of avian influenza on the northern gannet Colony of bass rock using ultra‐high‐resolution drone imagery and deep learning. Drones, 8(2), 40. 10.3390/drones8020040

[jane70145-bib-0038] Wong, C. (2024). Bird‐flu threat disrupts Antarctic penguin studies. Nature. 10.1038/d41586-024-00807-0 38491182

