# Supplementary material for: Estimating epidemiological parameters of highly pathogenic avian influenza in common terns using exact Bayesian inference
Source: J Anim Ecol. 2025 Oct 8;94(12):2491–503. doi: 10.1111/1365-2656.70145 (PMC12673236; doi:10.1111/1365-2656.70145)
Supplement: Supplementary file 1 — Table S1. The total PSRF values and total ESSs are shown for each parameter for the model without historically acquired immunity in 2022 and the model with historically acquired immunity in 2023. ’‐’ denotes that no value was calculated for this entry. Table S2. The posterior median (and 95% credible intervals) are shown for each parameter for the model with the “baseline” priors (main text Table 1), a prior giving a longer mean infectious period (μI ~ Γ(7, 10)), a prior giving smaller values to the shape parameters on the latent and infectious periods (kL , kI ~ Γ(2.5, 5)), and a prior giving a shorter latent period (μL ~ Γ(1, 10)). Results are shown for 2022 (top) and 2023 (bottom) separately with the DIC for each model. We multiply β1 by the colony size to facilitate comparison of the rates between years. Figure S1. The posterior distributions of the parameters inferred based on the Banter See data are shown by the coloured lines. The solid black lines showthe prior distributions. In the case of ph two prior distributions are shown because the priors differed for the two years. The solid coloured lines show estimates assuming the baseline level of underdetection given by the prior in Table 1 and the dashed lines show estimates based on a prior belief of a higher level of underdetection given by Tβ(11, 200, 0.16). Figure S2. The posterior distributions of the parameters inferred based on the Banter See data are shown by the coloured lines. The solid black lines showthe prior distributions. In the case of ph two prior distributions are shown because the priors differed for the two years. The solid coloured lines show estimates assuming density dependent transmission and the dashed lines show estimates assuming frequency dependent transmission. [file JANE-94-2491-s001.pdf]

Supplementary File: Estimating Epidemiological Parameters of Highly Pathogenic Avian Influenza in Common Terns using Exact Bayesian Inference

S1 Modified Gillespie Algorithm

We simulated from the model using a modified Gillespie algorithm as follows:

1. Initialise the time, $t = t_0$, and choose an initial externally exposed individual e_w.
2. Propose a set of exposure, infection and removal times, $\boldsymbol{e'}$, $\boldsymbol{i'}$, $\boldsymbol{r'}$, according to the assumed distributions of the latent and infectious periods and the current parameter values, $\boldsymbol{\theta^k}$.
3. Choose the earliest proposed time and update the corresponding set of exposure, infection or removal times, $\boldsymbol{e}$, $\boldsymbol{i}$, $\boldsymbol{r}$, to reflect this accepted time.
4. If the update in step 3 changed the number of infectious individuals then propose a new set of exposure times.
5. Repeat steps 3 and 4 until there are no exposed or infectious individuals remaining.

S2 Reversible jump Markov chain Monte Carlo algorithm

In practice, the posterior distribution is only known up to a constant of proportionality but samples from it can nonetheless be generated using a reversible jump Markov chain Monte Carlo (RJ MCMC) algorithm as follows:

1. **Initialisation:** $\boldsymbol{r}^{-}$ is set based on the observed mortality data. Sample an initial parameter set $\boldsymbol{\theta}$ from the prior $p(\boldsymbol{\theta})$. Create an initial set of exposure $\boldsymbol{e}$ and infection $\boldsymbol{i}$ times.
2. **Parameter updates:** A random walk Metropolis update is sequentially applied to each model parameter.

3. **State-space updates:** Make updates to $\boldsymbol{e}, \boldsymbol{i}, \boldsymbol{r}^+$ for fixed parameters $\boldsymbol{\theta}$ and initial infection time T_0. For computational efficiency changes are made to multiple individuals at once and the entire proposal is accepted with a Metropolis-Hastings acceptance probability. Because mixing in event-space tends to be slower than in parameter space, multiple state-space updates M are performed. This paper used $M = 40$.

4. **Update time of initial infection, T_0:** A random walk Metropolis update is performed on T_0, whilst fixing all other transition times and parameters $\boldsymbol{\theta}$.

5. **Jointly update T_0 and event times:** A random walk Metropolis update is performed on T_0, with a corresponding change in all other non-fixed event times, whilst fixing parameters $\boldsymbol{\theta}$.

6. **Store:** The current parameter set $\boldsymbol{\theta}$ is stored. Due to a large degree of correlation between successive iterations, this can potentially be done only every T iterations to save computational memory. This study used $T = 100$.

7. Repeat steps 2-6 over S iterations until the MCMC chain is well mixed.

S3 Parameter and state space updates

S3.1 Parameter updates

Parameter values are updated as follows. For a given parameter θ_i propose a new value θ_i' by drawing a value from a normal distribution with mean θ_i and variance σ_i^2. All other parameters in $\boldsymbol{\theta'}$ are set the same as in $\boldsymbol{\theta}$. The proposal is accepted with a Metropolis-Hastings probability

$$min\left(1, \frac{L(\boldsymbol{e},\boldsymbol{i},\boldsymbol{r}|\boldsymbol{\theta'})p(\boldsymbol{\theta'})}{L(\boldsymbol{e},\boldsymbol{i},\boldsymbol{r}|\boldsymbol{\theta})p(\boldsymbol{\theta})}\right), \tag{1}$$

in which case θ_i is set to θ_i', or else rejected. The proposal size σ_i is optimally tuned to give an acceptance rate of approximately 33% (see Section S4).

S3.2 State space updates

A state-space update consists of making m individual proposals (see below) to the state to generate $\boldsymbol{e'}$, $\boldsymbol{i'}$, $\boldsymbol{r^{+\prime}}$ and accepting this new event sequence with a Metropolis-Hastings probability

$$min\left(1, \frac{L(\boldsymbol{e'},\boldsymbol{i'},\boldsymbol{r}^{+\prime},\boldsymbol{r}^-|\boldsymbol{\theta})}{L(\boldsymbol{e},\boldsymbol{i},\boldsymbol{r}^+,\boldsymbol{r}^-|\boldsymbol{\theta})}\prod_m \frac{p_m^{p\to i}}{p_m^{i\to p}}\right). \tag{2}$$

Here m goes over the individual proposals and $p_m^{i\to p}$ represents the probability associated with each of these changes (note, $p_m^{p\to i}$ gives the proposal probability in the opposite direction).

For each iteration ($m = 1, ..., M$), when performing state space updates we select from the following nine proposal cases with the corresponding probabilities:

- **Case 1:** Convert a susceptible individual to an unobserved mortality.
  1. Randomly select j from n_S susceptible individuals (if $n_S = 0$ do not update).
  2. Sample an exposure time e'_j. This is done by setting up $B(= 100)$ equally sized bins spanning a period T from some time substantially before the outbreak until the end of the outbreak, T_f. p_b denotes an approximation to the probability of an exposure falling in the interval spanned by each bin. It is derived from an unnormalized vector P_b, which takes an initial uniform value V. P_b is dynamically updated after each full set of state-space proposals during the burn-in period. This update consists of incrementing P_b for every instance of an exposure event being contained within bin b. Consequently, as the burn-in period proceeds, so p_b evolves from its initial uniform distribution to one representing a better and better approximation to the posterior distribution in exposure time. The numerical value V was selected to be 100, which was found to allow for good convergence.
  3. Propose infection i'_j and removal r'_j times based on the gamma distributed latent and infected periods in the model.
  4. Set flags in the code to denote that this individual died but was unobserved.

  The proposal probability is given by

$$p_m^{i\to p} = \frac{1}{n_S} \times p_b \times \frac{B}{T} \times f_\gamma(i'_j - e'_j; \mu_L, k_L) \times f_\gamma(r'_j - i'_j; \mu_I, k_I). \tag{3}$$

  These five terms represent the probability of: selecting individual j, selecting bin b, sampling exposure time within bin b, sampling the latent period, and sampling the infectious period. The probability for the reverse transition $p_m^{p\to i}$ is calculated using Eq. (4).

- **Case 2:** Convert an unobserved mortality to a susceptible individual.
  1. Randomly select j from n_U unobserved mortalities (if $n_U = 0$ do not update j).
  2. Specify exposed e'_j, infection i'_j and removal r'_j times as unset.

  The proposal probability is given by

$$p_m^{i\to p} = \frac{1}{n_U} \tag{4}$$

and the probability for the reverse transition $p_m^{i\to p}$ is calculated using Eq.(3).

- **Case 3**: Convert a susceptible individual to a recovered individual. This is identical to case 1 except the flag should be set to denote that this individual recovered and was thus unobserved.

- **Case 4**: Convert a recovered individual to a susceptible individual. This is identical to case 2 except that we now have $p_m^{i\to p} = \frac{1}{n_R}$.

- **Case 5:** Update the exposure and infection times of an observed mortality.

  1. Randomly select j from n_O observed mortalities.
  2. If j is not the initial exposure:

     Propose a new set of exposure e'_j and infection i'_j times by sampling backwards from r_j based on the current distributions for the latent and infectious periods. If $e'_j < T_0$ individual j is not updated (where T_0 denotes the first exposure time). The proposal probability is given by

$$p_m^{i\to p} = \frac{1}{n_O} \times f_\gamma(i'_j - e'_j; \mu_L, k_L) \times f_\gamma(r'_j - i'_j; \mu_I, k_I). \tag{5}$$

  3. If j is the initial exposure:

     The exposure time remains unchanged $e'_j = e_j$ and we propose a new infection time $i'_j = e_j + \tau_j$ where $\tau_j \sim N(\mu_x, \sigma_x^2)$ and

$$\mu_x = \frac{(\mu_1\sigma_2^2 + \mu_2\sigma_1^2)}{\sigma_1^2 + \sigma_2^2} \qquad \text{and} \qquad \sigma_x^2 = \frac{\sigma_1^2\sigma_2^2}{\sigma_1^2 + \sigma_2^2} \tag{6}$$

     with $\mu_1 = e_j + \mu_L$, $\mu_2 = r_j - \mu_I$, $\sigma_1^2 = \frac{\mu_L^2}{k_L}$ and $\sigma_2^2 = \frac{\mu_I^2}{k_I}$. This corresponds to approximating the latent and infectious periods as normal (instead of gamma distributed) and taking the product of these. If i_j is not in the interval between e_j and r_j, individual j is not updated. The proposal probability is given by

$$p_m^{i\to p} = \frac{1}{n_O} \times f_N(i_j; \mu_x, \sigma_x^2). \tag{7}$$

- **Case 6**: Update either the exposure and infection times or the infection and removal times of an unobserved mortality with equal probability.

  Generate a random value from a uniform distribution between 0 and 1. If the number is less than 0.5 go to case (a) below, otherwise go to case (b).

  (a) 1. Randomly select j from n_U unobserved mortalities (if $n_O = 0$, individual j is not updated).

2. Propose a new set of exposure e'_j and infection i'_j times by sampling backwards from r_j based on the current distributions for the latent and infectious periods. If $e'_j < T_0$ individual j is not updated (where T_0 denotes the first exposure time).

The proposal probability is given by

$$p_m^{i \to p} = \frac{1}{n_U} \times f_\gamma(i'_j - e'_j; \mu_L, k_L) \times f_\gamma(r_j - i'_j; \mu_I, k_I). \tag{8}$$

(b) 1 Randomly select j from n_U unobserved mortalities (if $n_U = 0$, individual j is not updated).

2 Propose a new set of infection i'_j and recovery r'_j times by sampling forwards from e_j based on the current distributions for the latent and infectious periods.

The proposal probability is

$$p_m^{i \to p} = \frac{1}{n_U} \times f_\gamma(i'_j - e_j; \mu_L, k_L) \times f_\gamma(r'_j - i'_j; \mu_I, k_I). \tag{9}$$

- **Case 7**: Update either the exposure and infection times or the infection and removal times of a recovered individual, with equal probability. This is equivalent to Case 6 above but where we sample from n_R recovered individuals rather than n_U unobserved mortalities and replace n_U by n_R in the proposal probabilities.

- **Case 8**: Convert a susceptible individual to an individual with historical immunity with proposal probability

$$p_m^{i \to p} = \frac{1}{n_S} \tag{10}$$

The proposal probability for the reverse transition is given by Eq. 11. Set flags to denote this individual had historical immunity.

- **Case 9**: Convert an individual with historical immunity to a susceptible individual with proposal probability

$$p_m^{i \to p} = \frac{1}{n_H} \tag{11}$$

The proposal probability for the reverse transition is given by Eq. 10. Set flags to denote this individual did not have historical immunity.

We reflect that some events are more frequent than others by assigning the following probabilities of selecting each case: change exposure and infection times of observed individual (30%), add/remove a recovered individual (15% for each option), add/remove an unobserved mortality (2.5% for each option),

change exposure and infection or infection and removal time of unobserved infection (2.5% for each option), convert a susceptible individual to one with historical immunity or vice versa (15% for each option).

S3.3 Update time of initial infection, T_0

Propose an update, T_0', by sampling from a proposal distribution given by $N(T_0, \sigma_T^2)$ where $\sigma_T^2 = \frac{\mu_L^2}{k_L}$. Immediately reject this proposal if T_0' exceeds the first exposure time immediately following T_0. For all those individuals for which $e_j = T_0$ we set $e_j' = T_0'$ else $e_j' = e_j$. The proposal is accepted with probability

$$min\left(1, \frac{L(\boldsymbol{e'}, \boldsymbol{i}, \boldsymbol{r}^{+}, \boldsymbol{r}^{-}|\boldsymbol{\theta})}{L(\boldsymbol{e}, \boldsymbol{i}, \boldsymbol{r}^{+}, \boldsymbol{r}^{-}|\boldsymbol{\theta})}\right). \tag{12}$$

S3.4 Jointly update T_0 and event times

Propose an update, T_0', by sampling from a proposal distribution given by $N(T_0, \sigma_J^2)$ where σ_J^2. The proposal size σ_J is optimally tuned to give an acceptance rate of approximately 33%. For each individual j propose a new exposure time

$$e_j' = \begin{cases} T_0' & \text{if } e_j = T_0 \\ e_j + (T_0' - T_0)\left(\frac{\min(r_j, T_f) - e_j}{\min(r_j, T_f) - T_0}\right) & \text{otherwise} \end{cases} \tag{13}$$

and infection time

$$i_j' = \begin{cases} i_j + (T_0' - T_0)\left(\frac{\min(r_j, T_f) - i_j}{\min(r_j, T_f) - T_0}\right) & \text{if } i_j < T_f \\ i_j & \text{otherwise} \end{cases} \tag{14}$$

The proposed event sequence is accepted with a Metropolis-Hastings probability

$$min\left(1, \frac{L(\boldsymbol{e'}, \boldsymbol{i'}, \boldsymbol{r}^{+\prime}, \boldsymbol{r}^{-}|\boldsymbol{\theta})}{L(\boldsymbol{e}, \boldsymbol{i}, \boldsymbol{r}^{+}, \boldsymbol{r}^{-}|\boldsymbol{\theta})} \prod_j f_j^e f_j^e\right). \tag{15}$$

where the product j goes over all individuals and the factors

$$f_j^e = \begin{cases} 1 & \text{if } e_j = T_0 \\ 1 - \frac{T_0' - T_0}{\min(r_j, T_f) - T_0} & \text{otherwise} \end{cases} \tag{16}$$

and

$$f_j^i = \begin{cases} 1 - \frac{T_0' - T_0}{\min(r_j, T_f) - T_0} & \text{if } i_j < T_f \\ 1 & \text{otherwise} \end{cases} \tag{17}$$

account for the change in phase space as a result of this proposal.

S4 Details of the tuning procedure

For efficient operation of MCMC, proposals often need to be tuned. This procedure is performed during the burn-in period to ensure that detailed balance is strictly enforced when posterior samples are generated. The parameters which need to be tuned are: the sizes for parameter proposals σ_i, the size of the joint proposal on all non-fixed events σ_J and the number of individual updates per state update m. These are all treated in the same basic way. If a proposal is accepted the jumping size is multiplied by a factor u (bigger than one) and if rejected by a factor d (less than one). For σ_i and σ_J the values $u = 1.001$ and $d = 0.9995$ are used leading to an acceptance rate $\sim 33\%$. For m a floating point version m' is used (and m is calculated by rounding m' to the nearest integer above m) with $u = 1.001$ and $d = 0.999$, leading to an acceptance rate $\sim 50\%$ (this was found to be more efficient than $\sim 33\%$ due to a significant computational cost of undoing a proposal). Proposal values are initialised to essentially arbitrary values (for σ_i this is 10% of the mean of the prior, $\sigma_J = 1$ and $m' = 10$).

S5 Potential scale reduction factors and effective sample sizes

The potential scale reduction factors (PSRFs) and total effective sample sizes (ESSs) are shown for the model without historically acquired immunity in 2022 and the model with historically acquired immunity in 2023 (Table S1). These models were chosen because they have the lowest deviance information criterion values. Values for the other models presented in the main text were similar.

S6 Sensitivity to choice of latent and infectious period priors

Table S2 shows the posterior medians and 95% credible intervals for all model parameters under different priors for the latent and infectious period means and shapes to determine whether a different choice of informative priors would have a substantial impact on estimates of the parameter values of interest. Alternative values were chosen based on those reported by Kirkeby and Ward (2022). When assuming a shorter mean latent period of 1 day (rather than 2 days in the baseline model) we observed slightly reduced R_0 estimates in both 2022 and 2023. Increasing the prior on the mean infectious period from

Parameter	Year	PSRF	ESS
μ_L	2022<br>2023	1.002<br>1.001	167<br>604
k_L	2022<br>2023	1.002<br>1.000	330<br>4005
μ_I	2022<br>2023	1.002<br>1.002	187<br>641
k_I	2022<br>2023	1.002<br>1.000	354<br>3753
R_0	2022<br>2023	1.002<br>1.002	170<br>649
β_1	2022<br>2023	1.000<br>1.000	10479<br>13530
p_d	2022<br>2023	1.001<br>1.001	1072<br>1892
p_u	2022<br>2023	1.000<br>1.000	106276<br>3753
p_h	2022<br>2023	-<br>1.000	-<br>63099

Table S1: The total PSRF values and total ESSs are shown for each parameter for the model without historically acquired immunity in 2022 and the model with historically acquired immunity in 2023. '-' denotes that no value was calculated for this entry.

5 days to 7 days resulted in slightly increased R_0 estimates in both years as we would expect, though the credible intervals still overlapped those from the baseline model considerably. Decreasing the priors on the shape parameters had no notable impacts on any parameter estimates other than the shape parameters themselves. There was no notable impact of changing the latent or infectious mean priors on the external transmission rate, probability of historically acquired immunity or the probability of detection. The estimated probability that an infected individual died rather than recovered, p_d, was unaffected in 2022 but the upper limit of the credible interval did increase notably under a shorter latent period in 2023; however, the large DIC score would suggest that this model performed poorly. None of the choices of priors consistently gave the lowest DIC score across the 2 years. A mean latent period prior centered on 1 day gave the lowest DIC in 2022 but by far the highest score in 2023.

The estimated values in Table S2 suggest that our choice of priors is likely to be reasonable and the estimated R_0 and p_d values are likely to be quite robust to minor misspecification of the prior distributions.

S7 Impact of assumed underdetection

We tested how the prior placed on the probability that a mortality went undetected, p_u, influenced the posterior distributions of the other parameter values. Figure S1 shows that increasing the proportion of mortalities that are not detected had negligible impact on the posteriors for any of the other parameters. It is likely that when the probability of failing to observe carcasses becomes very high we would struggle

Parameter	2022<br>Baseline model	 Longer infectious	 Lower shapes	 Shorter latent
μ_L	2.0<br>(1.1; 3.5)	2.0<br>(0.9; 3.5)	2.1<br>(1.1; 3.4)	1.0<br>(0.5; 1.7)
k_L	4.7<br>(1.7; 10.3)	5.1<br>(1.9; 10.9)	2.7<br>(1.0; 5.5)	4.8<br>(1.6; 10.3)
μ_I	5.5<br>(2.9; 8.8)	6.7<br>(3.9; 10.4)	4.9<br>(2.8; 7.5)	5.4<br>(3.1; 8.4)
k_I	3.6<br>(1.6; 8.1)	3.7<br>(1.6; 8.4)	2.2<br>(1.0; 4.8)	4.3<br>(1.8; 9.6)
R_0	5.1<br>(3.0; 9.7)	5.9<br>(3.3; 12.5)	5.2<br>(3.2; 9.2)	3.7<br>(2.5; 6.1)
$\beta_1 N$	0.097<br>(0.001; 2.921)	0.087<br>(0.001; 3.111)	0.086<br>(0.001; 2.816)	0.071<br>(0.001; 2.071)
p_d	0.33<br>(0.28; 0.40)	0.33<br>(0.28; 0.39)	0.33<br>(0.28; 0.40)	0.34<br>(0.29; 0.41)
p_h	0.22<br>(0.12; 0.35)	0.21<br>(0.12; 0.33)	0.22<br>(0.12; 0.34)	0.21<br>(0.12; 0.33)
p_u	0.018<br>(0.005; 0.042)	0.018<br>(0.005; 0.042)	0.018<br>(0.005; 0.043)	0.018<br>(0.005; 0.042)
**DIC**	218,462	223,394	216,924	181,829
	**2023**			
μ_L	2.0<br>(0.8; 4.1)	2.4<br>(1.0; 4.3)	1.9<br>(0.8; 3.7)	0.9<br>(0.4; 1.6)
k_L	5.2<br>(2.2; 10.8)	5.2<br>(2.2; 10.8)	2.8<br>(1.2; 5.7)	4.8<br>(1.7; 10.4)
μ_I	4.0<br>(1.7; 8.1)	6.1<br>(2.3; 11.0)	3.5<br>(1.6; 6.7)	2.9<br>(1.5; 5.9)
k_I	5.2<br>(2.3; 10.5)	6.1<br>(2.8; 11.3)	2.9<br>(1.3; 5.7)	5.1<br>(2.1; 10.6)
R_0	3.2<br>(1.7; 7.0)	4.6<br>(2.1; 9.5)	3.1<br>(1.7; 6.2)	2.0<br>(1.3; 3.5)
$\beta_1 N$	0.237<br>(0.000; 3.509)	0.269<br>(0.000; 4.268)	0.140<br>(0.000; 3.160)	0.050<br>(0.000; 1.655)
p_d	0.14<br>(0.11; 0.20)	0.13<br>(0.10; 0.17)	0.14<br>(0.11; 0.20)	0.16<br>(0.12; 0.34)
p_h	0.13<br>(0.08; 0.18)	0.13<br>(0.08; 0.18)	0.13<br>(0.08; 0.18)	0.13<br>(0.08; 0.18)
p_u	0.018<br>(0.005; 0.043)	0.018<br>(0.005; 0.043)	0.018<br>(0.005; 0.043)	0.018<br>(0.005; 0.043)
**DIC**	66,407	39,517	61,135	250,338

Table S2: The posterior median (and 95% credible intervals) are shown for each parameter for the model with the "baseline" priors (main text Table 1), a prior giving a longer mean infectious period ($\mu_I \sim \Gamma(7, 10)$), a prior giving smaller values to the shape parameters on the latent and infectious periods ($k_L, k_I \sim \Gamma(2.5, 5)$), and a prior giving a shorter latent period ($\mu_L \sim \Gamma(1, 10)$). Results are shown for 2022 (top) and 2023 (bottom) separately with the DIC for each model. We multiply β_1 by the colony size to facilitate comparison of the rates between years.

to make robust inferences unless the population size was also very large because the amount of data remaining to run the inference on would become small. Nonetheless, provided the majority of carcasses are observed the ability to estimate the main parameters of interest appears robust to differing levels of underdetection.

S8 Frequency or density-dependent transmission?

We tested the impact of our assumption of frequency dependent as opposed to density dependent transmission by remaking Fig 4 from the main paper showing the posterior distributions for both transmission types (Fig S2). There is negligible difference in the posterior distributions between the two transmission modes. This is likely because the change in the colony size and thus the change in the density is relatively small over the duration of the outbreak. It is possible that a model that attempts to fully quantify the colony size at all times by accounting for the arrival and departure processes of the birds may find different results; however, this seems unlikely given that the majority of birds arrived before the outbreak began and the duration of the outbreak was relatively short (3-4 weeks). Consequently, it is likely that the main driver of change in colony size will be reduction due to mortality and this process is captured in the model. It should also be noted that if the outbreak started earlier in the season when a smaller proportion of birds had arrived and densities were lower then it is plausible that the difference between density and frequency dependent transmission would be later.

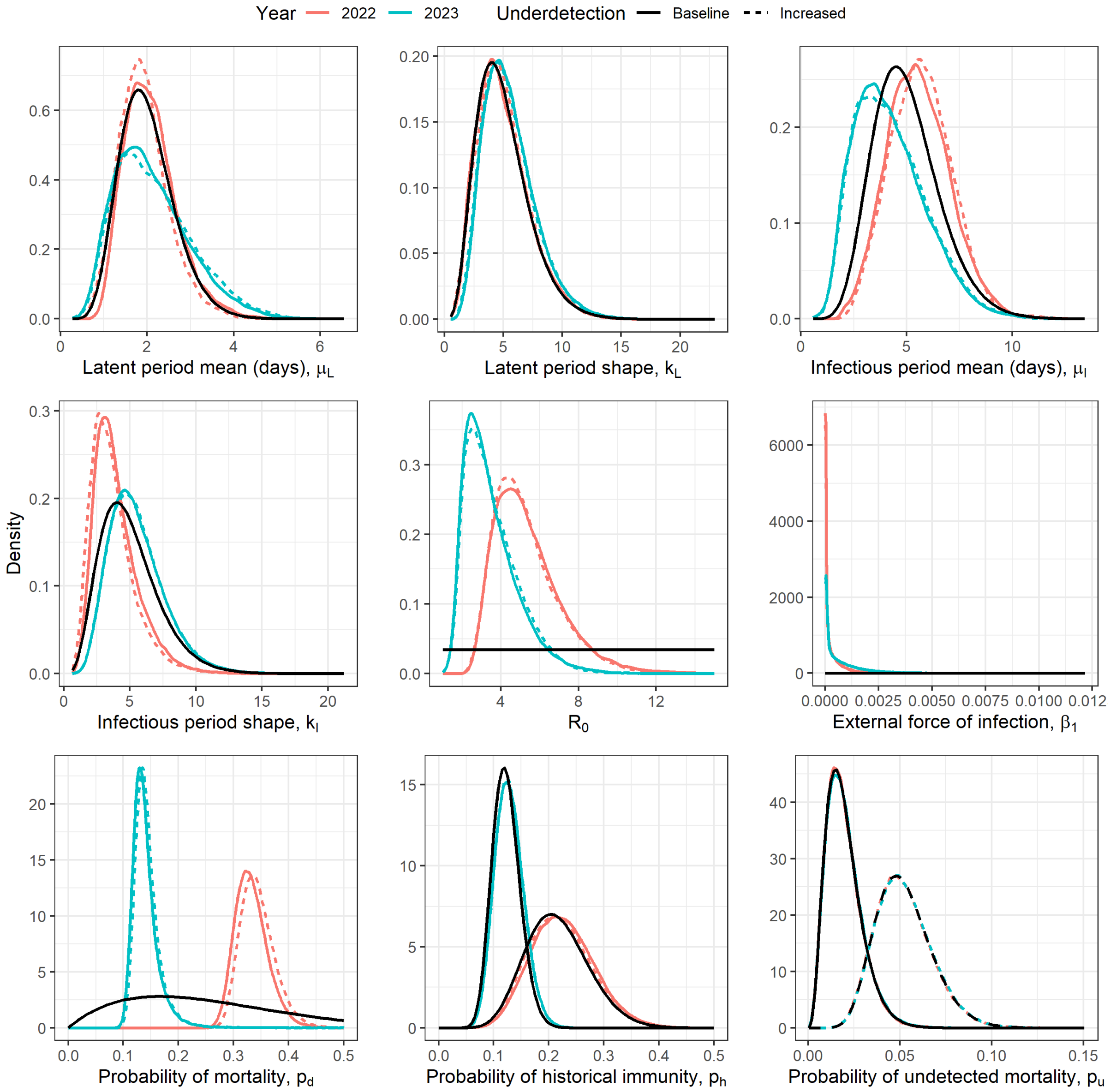

Figure S1: The posterior distributions of the parameters inferred based on the Banter See data are shown by the coloured lines. The solid black lines showthe prior distributions. In the case of p_h two prior distributions are shown because the priors differed for the two years. The solid coloured lines show estimates assuming the baseline level of underdetection given by the prior in Table 1 and the dashed lines show estimates based on a prior belief of a higher level of underdetection given by $T\beta(11, 200, 0.16)$.

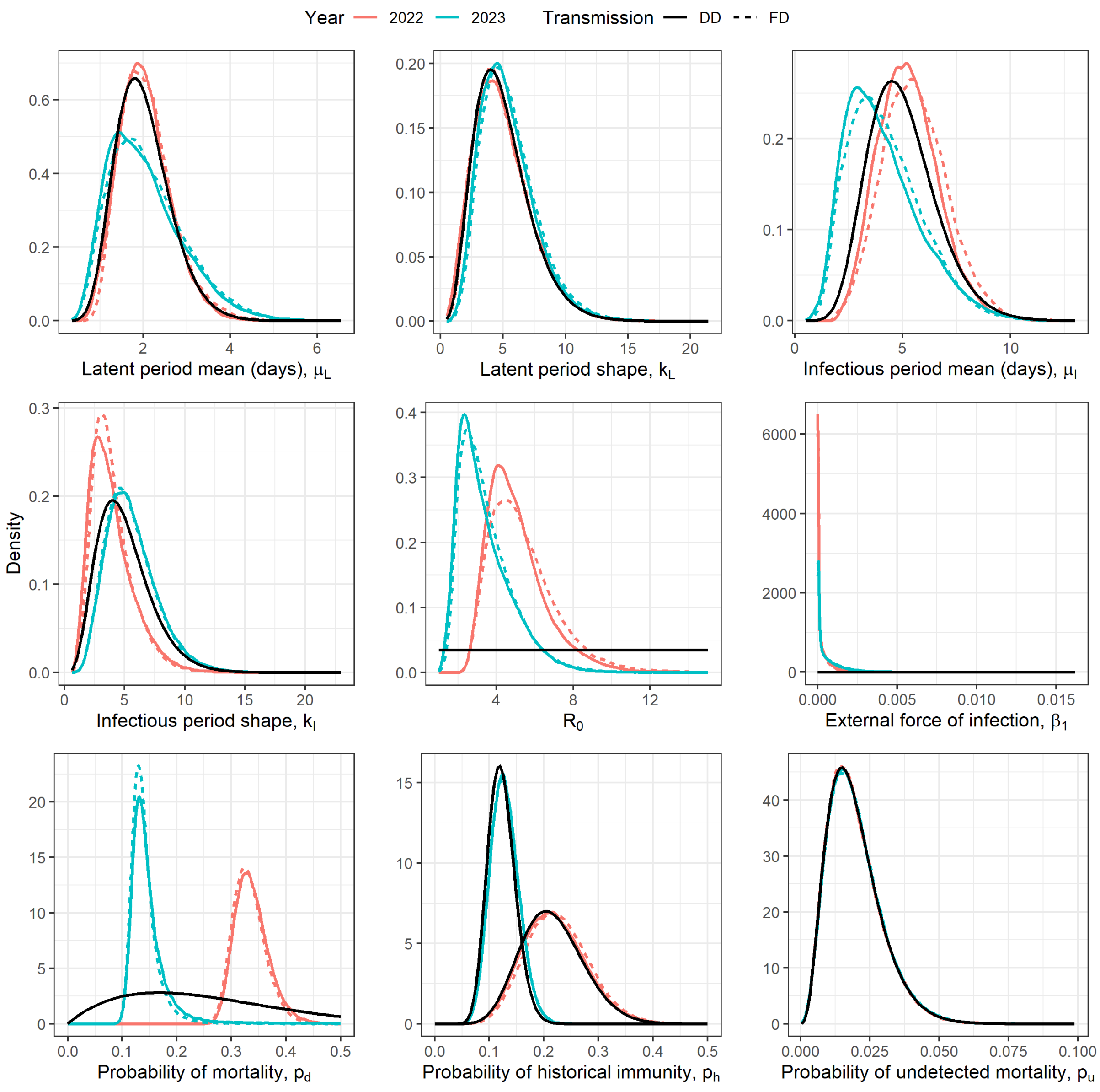

Figure S2: The posterior distributions of the parameters inferred based on the Banter See data are shown by the coloured lines. The solid black lines showthe prior distributions. In the case of p_h two prior distributions are shown because the priors differed for the two years. The solid coloured lines show estimates assuming density dependent transmission and the dashed lines show estimates assuming frequency dependent transmission.
